# Attenuation of the plasma volume response to crystalloid fluid used for goal-directed fluid therapy

**DOI:** 10.1186/s13613-025-01495-3

**Published:** 2025-06-19

**Authors:** Robert G. Hahn, Terry O’Brien

**Affiliations:** Karolinska Institutet at Danderyds Hospital (KIDS), Stockholm, Sweden

**Keywords:** Plasma volume, Crystalloid fluid, Colloid fluid, Pharmacokinetics

## Abstract

**Background:**

Goal-directed fluid therapy uses repeated bolus infusions of crystalloid or colloid fluid to increase the plasma volume for the purpose of challenging the patient’s position on the Frank-Starling curve. Each bolus is assumed to increase cardiac preload to the same degree. We examined whether this view is reasonable by simulating the plasma volume responses to crystalloid and colloid fluid. For this purpose, the volume kinetics of crystalloid fluid was characterized in 103 anaesthetized patients while parameters for colloid (hydroxyethyl starch) were taken from the literature. Simulations focused on the plasma volume response to 3 bolus infusions of 4 mL/kg of crystalloid and 2–4 mL/kg of colloid over 7 min. The boluses were separated by a free interval of 5 min to allow hemodynamic assessment.

**Results:**

Crystalloid fluid showed attenuation of the plasma volume response to repeated bolus infusions. The second and third bolus increased the plasma volume by only 51 and 36% as much as the first one. Attenuation also occurred when the boluses were preceded by a constant-rate infusion of 5 mL/kg/h or 10 mL/kg/h of crystalloid over 60 min, while placing the patient in the Trendelenburg body position (head down) reduced the attenuation. Bleeding increased the plasma volume responses, but attenuation still occurred. Colloid fluid did not show attenuation.

**Conclusion:**

Attenuation of the plasma volume response to bolus infusions of crystalloid fluid occurs. The second and third bolus might not increase cardiac preload enough to allow a correct diagnosis of fluid responsiveness. This problem is not shared by colloid fluid.

**Supplementary Information:**

The online version contains supplementary material available at 10.1186/s13613-025-01495-3.

## Introduction

Anesthetists administer fluids and drugs in surgery patients to ensure adequate systemic tissue perfusion pressure and oxygenation. Goal directed therapy (GDT) is a strategy designed to improve patient outcomes by optimizing cardiovascular dynamics and perfusion in the perioperative period. It involves monitoring key hemodynamic parameters like cardiac output (CO), blood pressure, oxygen saturation together with dynamic/static fluid preload responsiveness indices, and the stroke volume (SV) response to administered fluid. The goal for GDT is to optimize organ perfusion pressure and tissue oxygen delivery through targeted interventions that adjust fluid administration, vasopressors, and inotropes based on monitored parameters to achieve predefined goals [1].

Saugel et al. report that the most used GDT hemodynamic target variables are the dynamic cardiac preload variables [pulse pressure variation (PPV) or stroke volume variation (SVV)] and blood flow variables [2]. They point out that the term GDT is an imprecise umbrella definition that attempts to group differing hemodynamic treatment strategies together under the same term. However, at the core of most GDT protocols will be the requirement for individualized fluid administration. For example, protocols that target dynamic cardiac preload variables will require tailored fluid administration. Similarly, targeting blood flow will indicate the initial administration of fluids and then, if necessary, inotropes. Indeed, fluids may be the only intervention when the GDT target is to keep SVV below 10%, or to optimize/maximize SV in the immediate post induction period.

The GDT approach stems from the 1980 s and became a more viable therapeutic option with the introduction 25 years ago of less invasive hemodynamic monitors. Interest in GDT spread when evaluations showed that it reduced the number of postoperative complications [3]. However, the clear reduction of complication rate that was evident during the first years has become less clear with time [4–6]. This fall-off in impact of GDT might influenced by the increased adoption of surgical enhanced recovery protocols, more restrictive fluid management and the use of less invasive surgery.

These changes have no doubt improved surgical outcomes and made GDT outcome improvement studies more challenging. This study investigated whether another factor might need to be considered. Many surgical GDT protocols involve the use of repeated bolus infusions (fluid challenges) aimed to boost cardiac preload that, in turn, increases SV and CO. The fast infusion of fluid is intended to act as a fluid challenge to test for fluid responsiveness, which is defined as a SV response of ≥10%. If the SV response is less than 10%, the patient is deemed non-responsive and further no boluses are given. This process not only tests for fluid responsiveness but also can effectively move and leave the circulation on a more resilient and higher part on the Starling preload response curve.

A major change has occurred in a key component of this fluid challenge method. The use of colloids such as hydroxyethyl starch (HES) for the GDT fluid bolus infusions has been replaced by crystalloid fluid. This changeover occurred about a decade ago in the wake of reports of kidney injury associated with HES in septic patients [7]. The underlying, but unstated assumption, in the fluid challenge method is that delivering multiple boluses of a standardized volume of fluid will expand the plasma volume by a consistent amount across a sequence of such injections. Evaluating the percentage change in SV response for each individual bolus assumes a reproducible repeatable blood volume change has occurred, but variation between fluid types in terms of distribution losses could influence the resultant volume change. Pharmacokinetic issues were probably not immediately apparent when colloid was used for fluid challenges, as the loss of colloid from the circulation during and after each bolus will be quite small. Distribution losses of intravenous (IV) crystalloids on the other hand are known to be much faster. These losses may be of greater significance for variation in the plasma volume impact of fluid challenges when using crystalloid fluid [8].

While it is known that crystalloid and colloid fluid infusions distribute differently, the consequences of their different pharmacokinetic behavior for fluid challenges are difficult to grasp without computer simulation. The present study explores, with use of a pharmacokinetic model derived from prior fluid administration studies, the effect of standardized fluid bolus infusions on the resulting plasma and blood volume expansion. Both crystalloid and colloid fluid models were used for comparison. The primary hypothesis was that the volume expansion resulting from multiple bolus infusion during surgery is dependent on the pharmacokinetic profile of the fluid used. The related secondary hypothesis was that the use of crystalloid for fluid challenges will result in lower peak and more variability between sequential fluid challenge plasma volume levels than experienced when using colloid. The impact of maintenance fluid infusions and surgical hemorrhage on the plasma volume impact of fluid challenges were also studied.

## Methods

Material for an analysis of crystalloid fluid kinetics during general anesthesia was derived from a database of IV infusion experiments in humans that were performed using similar protocols. They were all planned and supervised by one of the authors (RGH). For the current presentation, 103 experiments were included where approximately 1.5 L of Ringer´s acetate/lactate was infused over 30–60 min. The data included 12 laparoscopic cholecystectomies [9], 30 laparoscopic gynecologic resections [10, 11], 29 thyroid surgeries [12], 7 open gastrointestinal operations [13], and 25 open hysterectomies [14] **(**Table 1**).** The cholecystectomies were performed with the patient placed in the reverse Trendelenburg position (20° head up) and the laparoscopic gynecological operations in the Trendelenburg position (approximately 30° head down), while the patients remained in the flat recumbent position during the other surgeries. Two studies randomized patients between IV and inhaled anesthesia [11, 13] while the others used both types of anesthesia. One patient in the thyroid group was excluded due to major hemorrhage (1.7 L) and one patient in the hysterectomy group was also excluded for the same reason, while being replaced by a subsequent patient.

**Table 1:** Details on the studies on which the population kinetic analuysis was applied

Operation	N	Body position	Body weight (kg)	Age (years)	Infused volume (L)	Infusion time (min)	MAP during study (mmHg)	Study period, mean (range)
Laparoscopic cholecystectomy	12	Reverse Trendelenburg (head up)	78 ± 8	44 ± 12	1.56 ± 0.17	55 ± 7	72 ± 12	90 (55–70)
Thyroid surgery	29	Flat recumbent	68 ± 11	53 ± 16	1.70 ± 0.28	30	70 ± 14	150
Laparoscopic gynecologocal	30	Trendelenburg (head down)	56 ± 9	40 ± 12	1.24 ± 0.30	42 ± 15	82 ± 14	124 (90–180)
Open hysterectomy	25	Flat recumbent	75 ± 13	47 ± 15	1.87 ± 0.34	30	85 ± 14	91 (90–120)
Open abdominal	7	Flat recumbent	70 ± 10	57 ± 12	1.75 ± 0.25	45	67 ± 17	83 (70–130)

Exclusion criteria were emergency surgery, regional anesthesia, age < 18 years, pregnancy, severe allergy, expected blood loss > 500 mL, and American Society of Anesthesiologist’s (ASA) physical status classes III–IV. Hence, no patient had severe cardiovascular disease or decreased kidney function. No subject underwent a dehydration procedure, blood withdrawal (in addition to sampling volume), or received an adrenergic drug as part of the protocol. Surgical hemorrhage during the period of data collection was minimal (50–150 mL) and no hemorrhage occurred before the study started.

### Data collection

Patients were in the fasting state and either received no premedication or diazepam by mouth. No fluid was administered during the induction of anesthesia but was initiated when the surgery was to begin. The hemoglobin (Hb) concentration venous blood was sampled in 3-mL aliquots every 5 min during the infusion, for 30 min thereafter, and then at 10–15 min intervals. Hb was analyzed at the same hospital´s clinical chemistry laboratory with a coefficient of variation of 0.7–1.0%. Data obtained after awakening from anesthesia were excluded.

The plasma dilution was calculated as [(Hb_baseline_/Hb_later_) − 1)]/(1 − hematocrit_baseline_). This equation is derived elsewhere [15]. Each dilution underwent a minimal mathematical correction to account for surgical hemorrhage, if any, and blood sampling [12].

Non-invasive mean arterial pressure (MAP) was measured in the arm not used for fluid infusion using an automatic device (Datex-Ohmeda, Box 900, Finland).

### Kinetic model

Population (mixed models) kinetics is the standard procedure based on likelihood mathematics that is used for quantifying the disposition of drugs and their association with drug effects in humans. The principle is to collect data on the concentration of the drug in a body fluid, usually the plasma, of many subjects and then to mathematically fit a kinetic model to these data as if only one person was studied. Characteristics between subjects or groups that affect the kinetic parameters, such as sex and body weight, are quantified by covariates. The output from the analysis consists of parameters determining the rates of distribution of the drug between the body spaces (compartments) that harbor the drug [16]. All marketed drugs are required to be characterized according to these principles.

Volume kinetics is an adaptation of drug kinetics that allows infusion fluids to be studied. The compartment walls in drug kinetics do not change with the dose, while their expansion constitutes the therapeutic effect of an infusion fluid. The expansion is created by an infusion-induced increase of the plasma water concentration, which is a dilution effect that can most easily be quantified by measuring blood hemoglobin concentration [17].

Studies of volume kinetics support that the flows of fluid between body fluid spaces represent mono-exponential functions, i.e., the flow is directly proportional to the volume expansion. Nearly all biological processes follow this kinetics, exceptions being receptor binding and Michaelis–Menten kinetics which are not applicable here. Hence, the distribution of infused fluid from the plasma to the interstitium occurs twice as fast if the plasma volume expansion is doubled, while the rate gradually decreases along with the decrease of the volume expansion. This conclusion is based on serial analysis of the blood hemoglobin (Hb) concentration and the urine output on during hundreds of infusion experiments.

**Fig. 1:**
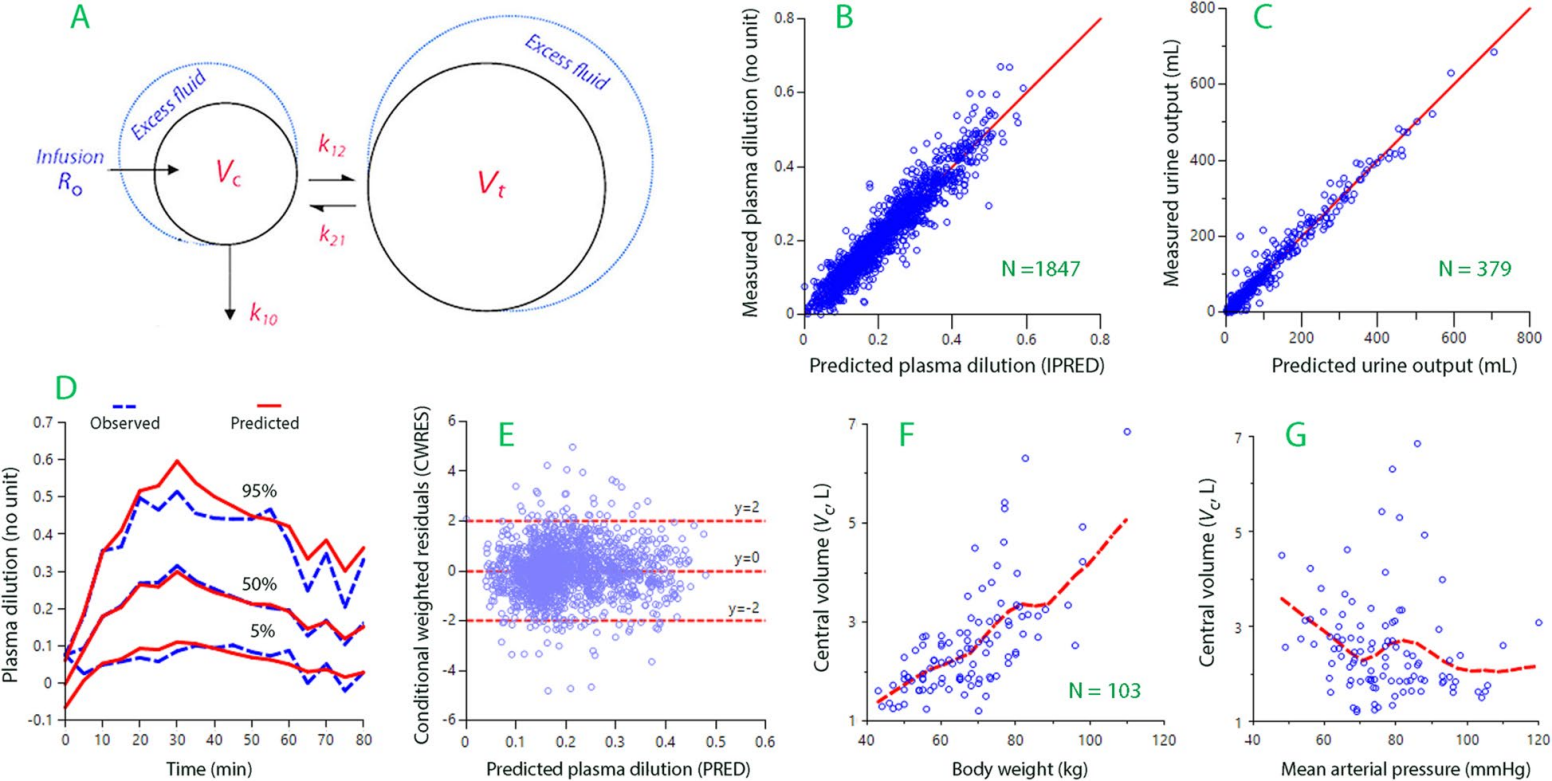
Kinetic model and goodness-of-fit. **A** Schematic drawing of the kinetic model used in the study. **B** Measured vs. model-predicted plasma dilution (N = 1847). **C** Measured vs. model-predicted urine output (N = 379). **D** Predictive check based on 1000 simulations. The plot allows visual comparison between the observed confidence intervals and the one predicted by the final model. **E** Conditional weighted residuals vs. the predicted plasma dilution without considering of the covariates. Few data points outside ± 3 SD signify a good model. **F** Increase of *V*_c_ with the body weight. **G** Decrease of *V*_c_ with the mean arterial pressure (MAP), which suggests that a low MAP allowed accelerated inflow of lymph to the plasma [34]

Recent studies show that appropriate volume kinetic model for crystalloid fluid (1-volume, 2-volume, or 3-volume) is dependent on the infused volume. The two-volume model describes the distribution of fluid between two fluid spaces, *V*_c_ (central) and *V*_t_ (peripheral) should be used when 500–1500 mL is administered (Fig. 1A) [18]. The “third fluid space” in humans opens for filling a bit later in the anesthetized than in the awake state and, therefore, the two-volume volume kinetic model was chosen.

In the two-volume model, fluid is infused at the rate *R*_o_ into the plasma, *V*_c_, from which distribution occurs at a rate governed by a rate constant *k*_12_ to the interstitial space, *V*_t_. Distributed fluid returns to *V*_c_ at a rate determined by a constant *k*_21_. Urinary excretion (U) occurs in proportion to the expansion of *V*_c_ by a rate constant *k*_10_. Here, *k*_10_ was obtained as the measured urinary excretion divided by the area under the curve for the central volume expansion (*v*_c_ − *V*_c_) for the period during which urine was collected.

The mathematical solutions to the following differential equations were used to predict the distribution of the infused fluid volume:

$${\text{d}}v_{{\text{c}}} /{\text{dt}} = R_{{\text{o}}} {-}k_{12} (v_{{\text{c}}} {-}V_{{\text{c}}} ) + k_{21} (v_{{\text{t}}} {-}V_{{\text{t}}} ){-}k_{10} (v_{{\text{c}}} {-}V_{{\text{c}}} )$$

$${\text{d}}v_{{\text{t}}} /{\text{dt}} = k_{12} (v_{{\text{c}}} {-}V_{{\text{c}}} ){-}k_{21} (v_{{\text{t}}} {-}V_{{\text{t}}} )$$

$${\text{dU}}/{\text{dt}} = k_{10} (v_{{\text{c}}} {-}V_{{\text{c}}} ).$$

Baseline parameters are denoted by capital letters and expanded volumes by lower-case letters (*v*_c_, *v*_t_). Volume expansion of the central fluid space, (*v*_c_ − *V*_c_) was indicated by the plasma dilution, which equals (*v*_c_ − *V*_c_)/*V*_c_. The glycocalyx water is part of *V*_c_, which is a quasi-measure of the plasma volume [19].

The equations show that the rate constants (*k*_12_, *k*_21_, and *k*_10_) determine the rates of mono-exponential functions because the flow rates are directly proportional to the volume expansion from where the flow begins.

### Covariates

The model parameters could be modified by covariates. In the present study, the examined covariates were age, sex, body position, MAP, infused volume, and infusion rate. Identification of which covariate affected which kinetic parameter was guided by plots of random effects as given by the “eta vector” as provided by the program used to analyze the kinetics. The most promising candidate parameters were tested, one by one, by adding them to the base model in a diagonal design. Two covariate models were used: The *exponential covariate model* was used to analyze the influence of categorical variables on the parameters in the base model. An example from Table 2 is that the rate constant *k*_12_ had a base value of 0.0682 and covariate effect −1.30 when the patient was placed in the Trendelenburg body position (head down). The value for *k*_12_ in the head down position then becomes:

$$0.0{682}*\left[ {{\text{e}}^{{{\text{flat recumbent }} = \, 0,{\text{ head down }} =\, {-1.30}}} } \right]$$

*k*_12_ collapses to the group value of 0.0682 min^−1^ when the patient is placed in the flat recumbent position (e^0^ = 1). By contrast, *k*_12_ in the head down position becomes 0.0682*(2.718^−1.30^) = 0.0186, i.e., 27% of the value obtained in the flat recumbent position.

**Table 2:** Population kinetic parameters for infused fluid volume in the final model

Kinetic parameter	Covariate	Covariate model	Best estimate	95% CI	CV%	−2 LL
tv*V*_c_ (L)			3.31	2.56–40.6	11.5	
tv*k*_12_ (10^–3^ min^−1^)			68.2	53.8–82.6	10.7	
tv*k*_21_ (10^–3^ min^−1^)			19.9	15.5–24.3	11.4	
tv*k*_10_ (10^–3^ min^−1^)			2.08	1.61–2.56	11.6	−1043
*V*_c_	0–10 min	Exponential	−0.34	−0.42 to −0.27	−11.3	
	10–20 min	Exponential	−0.19	−0.25 to −0.14	−14	−1250
*V*_c_	Body weight	Power	1.12	0.84–1.41	13.0	−1301
*k*_21_	Head down	Exponential	−1.10	−1.73 to −0.48	−29.0	
	Head up	Exponential	−1.45	−2.08 to −0.81	−22.4	−1339
*k*_12_	Head down	Exponential	−1.30	−1.63 to −0.97	−12.9	
	Head up	Exponential	−0.44	−0.68 to −0.20	−27.6	−1411
*V*_c_	MAP	Power	0.35	0.19–0.51	22.8	−1510
*V*_c_	Female sex	Exponential	−0.31	−0.54 to −0.08	−37.5	−1516
Full block model	All of the above					−1556
*Equations*						
$$\begin{gathered} V_{{\text{c}}} = { 331}0\left[ {({\text{body weight }}/{ 67}.{2})^{{{1}.{12}}} ]^{\phantom{a} } [({\text{MAP }}/{ 76}.{3})^{{0.{35}}} ]^{ \phantom{a}} [{\text{e}}^{{{\text{male}} = 0,{\text{ female}} = \, - 0.{31}}} ]} \right]^{ } \left[ {{\text{e}}^{{{2}0 - {18}0{\text{ min}} = 0, \, 0 - {1}0{\text{ min }} = \, - 0.{34},{1}0 - {2}0{\text{ min }} = \, - 0.{19}}} } \right]^{ } \hfill \\ k_{{{12}}} = \, 0.0{682}\left[ {{\text{e}}^{{{\text{flat}} = 0,{\text{ head down}} = \, - {1}.{3}0,{\text{ head up}} = \, - 0.{44}}} } \right] \hfill \\ k_{{{21}}} = \, 0.0{199}\left[ {{\text{e}}^{{{\text{flat}} = 0,{\text{ head down}} = \, - {1}.{1}0,{\text{ head up}} = \, - {1}.{45}}} } \right] \hfill \\ \end{gathered}$$						

Shown are the typical values (tv) for the fixed parameters in the group, followed by individual-specific covariates. The complete equations for the model parameters affected by covariates are given below

*tv* typical value for the group. *CI* confidence interval, *CV*% coefficient of variation (inter-individual)

*LL* log likelihood for the model during development. Mean body weight 67.2 kg, Mean MAP 76.3 mmHg

*Power model* was used for the relationship between MAP and *V*_c_. The group value was 3.31 L, the mean MAP 76.3 mmHg, and the covariate effect 0.35. At a MAP of 100 mmHg, *V*_c_ becomes:

$$3.31*\left[ {\left( {100/76.3} \right)^{0.35} } \right] = 2.32{\text{ L}}$$

When several statistically significant covariates were identified, they were applied to the model in a multivariate fashion. How these corrections based on covariate effects mathematically affect all parameter values are illustrated at the bottom of Table 2**.**

### Kinetic analysis

The final kinetic parameters and the covariate values were obtained by fitting the kinetic model to all measurements of plasma dilution and urinary excretion (dependent variables) in a single run using the Phoenix software version 8.3.4 for nonlinear mixed effects (Pharsight, St. Louis, MO) with the First-Order Conditional Estimation Extended Least Squares (FOCE ELS) as search routine and the additive error model. The criterion for accepting a covariate was that its inclusion should reduce the −2 LL (log likelihood) for the model by > 3.84 points (*P* < 0.05). Moreover, the 95% confidence interval (CI) for the estimate of the covariate was not allowed to include 0.

### Simulations

Prediction of the plasma volume expansion resulting from 3 consecutive 7-min bolus infusions were made using the simulation function in Phoenix after entering the optimal estimate of each kinetic parameter. A “free” interval of 5 min was added between the bolus infusions to allow time for a hemodynamic measurement to be made. The choice of the 7-min infusion time was chosen because a shorter time could be difficult to carry out. Moreover, the same set-up was used in one of our clinical trials [20].

The simulated infusion volume was 4 mL/kg, which was considered effective challenge of cardiac preload by Aya et al. [21] Half that volume (2 mL/kg) of colloid was simulated as this is the approximate short-term volumetric difference between Ringer´s solution and HES [22, 23].

The simulations that involved hemorrhage were treated by first simulating the filling of *V*_c_ and *V*_t_ at the end of a 60-min constant-rate infusion. The plasma volume lost with the hemorrhage was then subtracted from the simulated expansion of *V*_c_ whereafter the plasma volume responses to the three bolus infusions were simulated. The baseline was moved if the plasma loss with the hemorrhage exceeded the filling of *V*_c_ at the end of the constant-rate infusion, which means that *V*_c_ was set to zero mL while the baseline shift was added to the starting volume in *V*_t_. However, in the latter case the patients were hypovolemic, and the kinetic parameters changed according to that situation. Kinetic data for hypovolemic states is only available for conscious volunteers and, therefore, the “best evidence” was believed to be obtained by applying the linear covariate effects for blood withdrawal in awake volunteers to the parameters estimated here; these analyses were both made using same two-volume model. These covariate effects were obtained by infusing 25 mL/kg of Ringer´s solution in healthy volunteers in random order on three separate occasions. On one of these occasions no blood was withdrawn. On a second occasion 450 mL was withdrawn immediately before the infusion was started. On the third occasion 900 mL was withdrawn immediately before fluid was infused. The linear covariance was used to estimate the influence of the blood losses (400 and 600 mL) on the kinetic parameters used as examples in the present study [24].

The kinetic data for colloid fluid was taken from a previous study of hydroxyethyl starch 130/0.4 (HES) [25]. The intravascular persistence time was 116 min, which is the essential parameter for the degree of attenuation of the plasma volume response to a colloid fluid. For comparison, a recent study of gelatin (Gelofusine) during vascular surgery yielded a persistence time of 115 min when the influence of norepinephrine on the parameters had been calculated off (norepinephrine shortens the intravascular persistence time) [26].

### Statistics

Data showing a normal distribution are reported as the mean ± standard deviation (SD). Kinetic parameters are reported as the best estimate and 95% CI according to the output from the Phoenix program. Significance levels for inclusion of covariates were taken from the Phoenix program.

## Results

### Demographics, kinetic analysis

The 103 anesthetized patients were aged 47 ± 13 years, had body weight of 67 ± 13 kg, and blood Hb of 119 ± 12 g/L at baseline. The majority were women (91%). They received 1592 ± 379 mL of crystalloid fluid during 37 ± 12 min (range 30–60 min) and were monitored during 116 ± 31 min. MAP was 77 ± 14 mmHg when the infusions started (Table 1**).**

The kinetic analysis was based on 1,847 measurements of plasma dilution and 379 of urine output (mean, 17.9 and 3.7 per operation).

The final parameters in the kinetic analysis are shown in Table 2 from where the optimal values were used for later simulations.

Goodness-of fit measures are shown in Fig. 1B–E and covariances in Fig. 1F, G. The original data is given in the Supplementary File.

### Simulations of crystalloid versus colloid fluid

All simulations showed the plasma volume expansion resulting from three consecutive fluid boluses infused over 7 min and separated a by a free interval of 5 min. Each bolus infusion of crystalloid amounted to 4 mL/kg and each colloid bolus to 2 mL/kg.

**Fig. 2:**
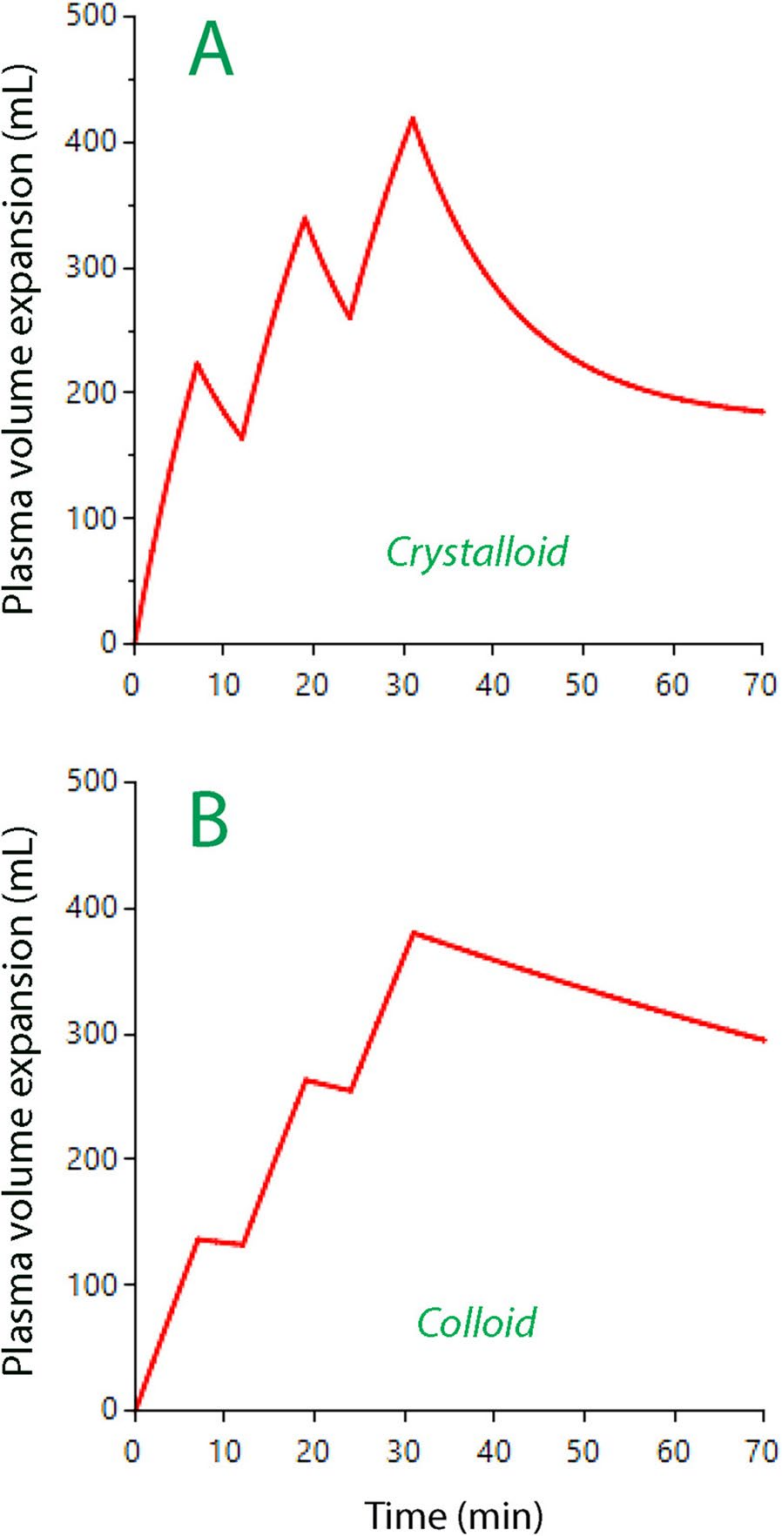
Simulation of the plasma volume expansion following three successive bolus infusions during 7 min followed by a free interval of 5 min in a patient weighing 70 kg. **A** Crystalloid fluid 4 mL/kg in each bolus (Ringer solution). **B** Colloid fluid 2 mL/kg in each bolus (hydroxyethyl starch)

The results show that a crystalloid fluid induces a smaller plasma volume expansion for each new bolus infusion**.** The increase of the plasma volume at the end of the second and third bolus would only be 51 and 36% as great as for the first one (Fig. 2A**)**.

By contrast, the plasma volume expansion resulting from the second and third bolus infusions of colloid fluid were 92 and 86%, respectively, of the first bolus (Fig. 2B).

### Constant-rate infusion

**Fig. 3:**
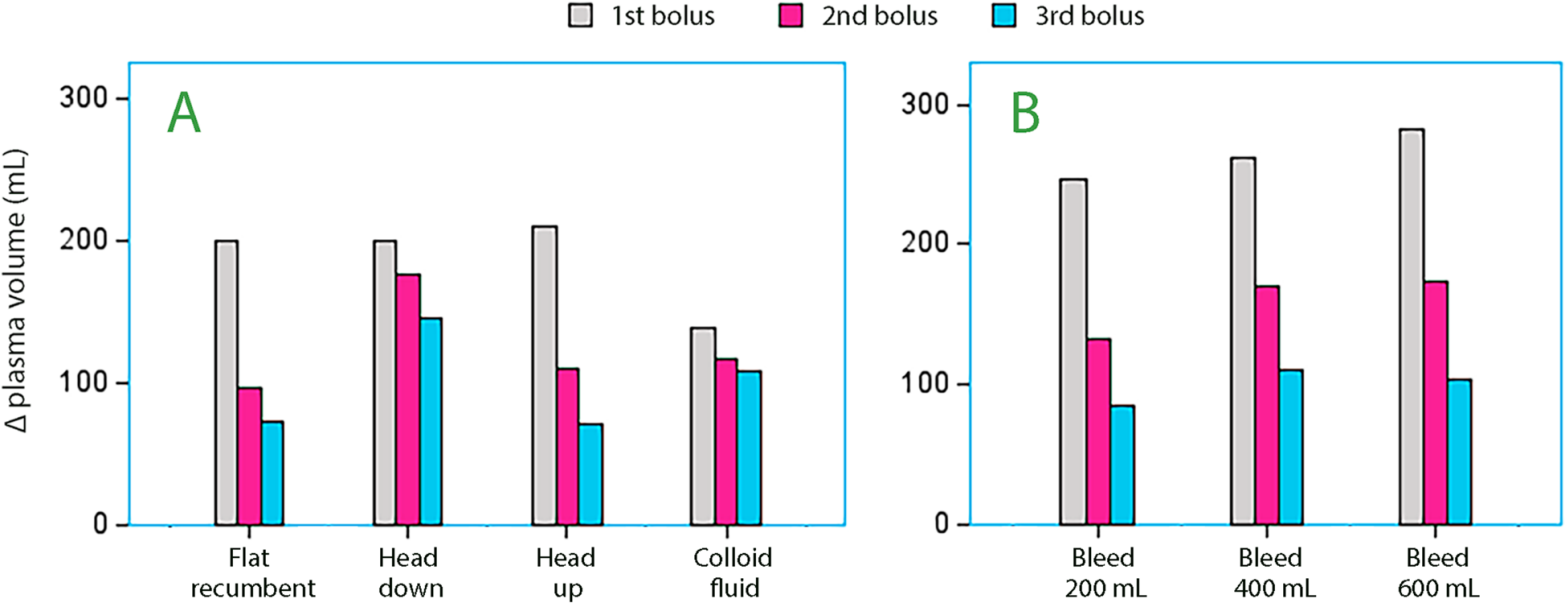
Simulation of the plasma volume expansion following three successive bolus infusions during 7 min followed by a free interval of 5 min in a patient weighing 70 kg after a constant-rate infusion 5 mL/kg/h of crystalloid fluid infused over 60 min. **A** Crystalloid fluid 4 mL/kg in each bolus depending on body position and 2 mL/kg of colloid fluid in the flat recumbent position. **B** Bolus infusions of crystalloid fluid given a hemorrhage suddenly occurs at 60 min.

**Fig. 4:**
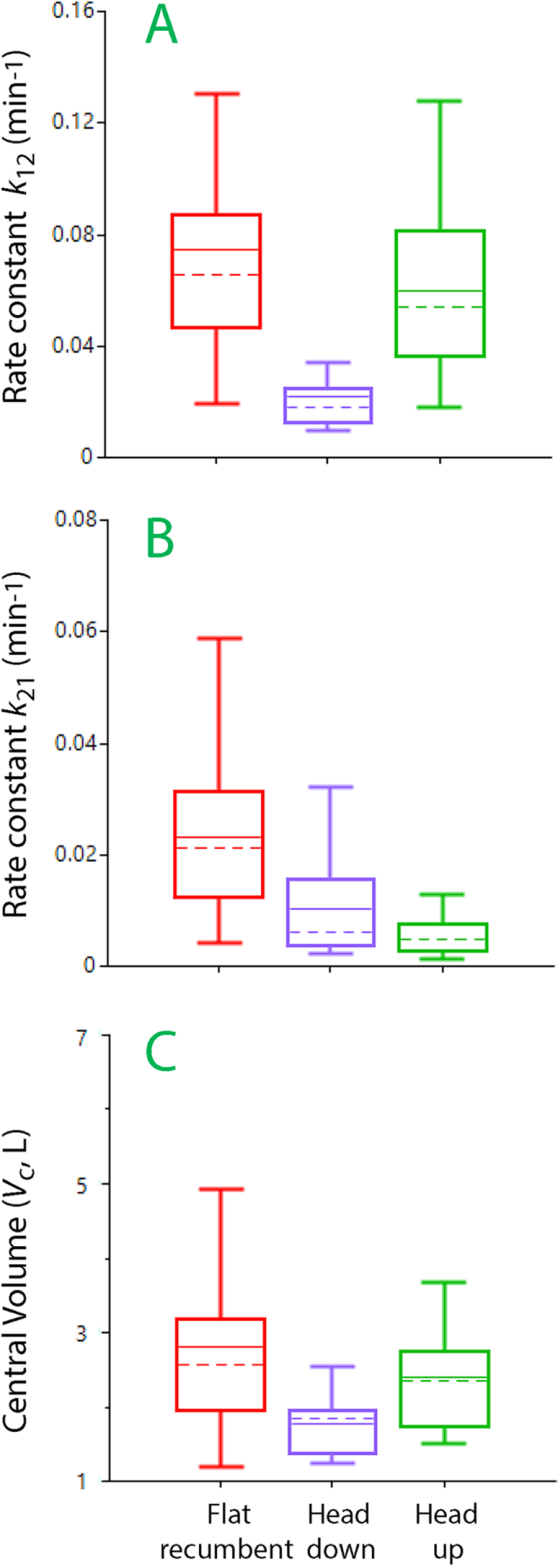
Kinetic differences depending in body position during general anesthesia. **A** The distribution rate constant (*k*_12_). **B** The rate constant for the return flow to the plasma (*k*_21_, lymphatic flow). **C** The size of the central fluid space at baseline (*V*_c_). The low *k*_12_ for the “head down” and the low *k*_21_ for the “head up” position are probably due to gravitational forces, while the unrealistically low *V*_c_ for “head down” is likely due to accelerated inflow of lymph [34]. The covariate analysis aligned these three differences with the data for the flat recumbent position

Simulations were performed where the three bolus infusions were preceded by a constant-rate infusion of 5 or 10 mL/kg/h of Ringer´s solution over 60 min. In the flat recumbent body position, these fluid challenges showed attenuation in the same way as when tested without preceding constant-rate infusion. However, a patient placed in the Trendelenburg position (head down) would have a similar plasma volume expansion in response to all three bolus infusions (Fig. 3A) which was due to depression of the distribution rate parameter (*k*_12_) (Fig. 4). Patients in the reverse Trendelenburg position (head up) would show the same decrease in volume effect for each bolus as in the flat recumbent position.

By contrast, colloid fluid showed a consistent plasma volume response when preceded by constant-rate infusions of crystalloid (Fig. 3A).

More data on these simulations is given in Table 3**,** which also shows the output when the boluses were preceded by 60-min constant-rate infusions crystalloid fluid.

**Table 3:** Simulations of the plasma volume expansion resulting from three consecutive bolus infusions of either 4 mL/kg crystalloid fluid or 2 mL/kg of colloid over 7 min separated by a free interval of 5 min in an anesthetized subject weighing 70 kg

Fluid	1 st bolus	2nd bolus	3rd bolus	ΔPlasma volume 1 st-2nd-3rd bolus
Crystalloid (3 × 4 mL/kg bolus)				
Flat recumbent				
0	0–224	165–339	260–419	224; 115; 80
5 mL/kg/h	126–326	257–423	341–496	200; 97; 73
10 mL/kg/h	251–428	349–507	421–573	177; 79; 66
Trendelenburg				
0	0–261	236–468	425–636	261; 207; 168
5 mL/kg/h	214–452	413–628	575–773	199; 176; 145
10 mL/kg/h	428–643	590–788	725–911	215; 145; 123
Reverse Trendelenburg				
0	0–240	192–382	308–471	240; 142; 89
5 mL/kg/h	131–341	277–451	369–522	210; 110; 71
10 mL/kg/h	262–443	363–521	421–574	181; 78; 53
Hydroxyethyl starch (3 × 2 mL/kg bolus)				
0	0–137	132–263	255–381	137; 126; 118
Ringer 5 mL/kg/h, then starch	126–265	256–381	369–489	139; 116; 108
Ringer 10 mL/kg/h, then starch	251–378	365–486	470–586	127; 108; 100

The bolus infusions could be preceded by no fluid (0) or by a continuous-rate infusion of crystalloid fluid over 60 min amounting to 5 mL/kg/h or 10 mL/kg/h. No blood loss was modeled

### Hemorrhage

Hemorrhage induced after a 60-min continuous-rate infusion of crystalloid fluid resulted in a strong initial plasma volume response to the first bolus but was gradually attenuated with repeated infusions (Fig. 3B). The hypovolemic state resulted in slightly stronger filling of the plasma volume, but differences depending on whether the constant-rate infusion amounted to 5 mL/kg/h or 10 mL/kg/h were slight **(**Table 4**).**

**Table 4:** Simulations of the plasma volume expansion resulting from three consecutive bolus infusions of 4 mL/kg crystalloid fluid over 7 min separated by a free interval of 5 min in an anesthetized subject weighing 70 kg

Fluid	Blood loss (mL)	1 st bolus	2nd bolus	3rd bolus	ΔPlasma volume 1 st-2nd-3rd bolus
Blood loss and normovolemia					
Ringer 5 mL/kg/h	200	0–246	196–378	302–462	246; 132; 84
Ringer 10 mL/kg/h	200	121–367	317–499	423–584	246; 132; 85
Blood loss, and hypovolemia					
Ringer 5 mL/kg/h	400	(−135)–127	92–297	232–408	262; 170; 111
	600	(−265)–18	(−12)–191	124–295	283; 173; 104
Ringer 10 mL/kg/h	400	(−9)–259	227–434	371–548	268; 175; 114
	600	(−139)–133	98–296	227–396	272; 163; 100

All bolus infusions were preceded by a continuous-rate infusion of crystalloid fluid at 5 mL/kg/h or 10 mL/kg/h over 60 min. Various blood losses without causing hypotension were incorporated into the kinetic model. Hct was 35%

### Exploratory analyses

We also simulated constant-rate infusions lasting 30, 90, and 120 min before the bolus infusion were initiated (in addition to the 60-min duration). They merely changed the starting point for the bolus infusions, which magnitudes and patterns remained unchanged.

Table 5 highlights the differences in plasma volume expansion, blood volume expansion, and the estimated capillary leakage rate when *identical* amounts of colloid and crystalloid fluid are used in a single 7-min fluid challenge.

**Table 5:** Plasma volume expansion, blood volume expansion, and capillary leakage rates at the end of a single 7-min fluid challenge consisting of 4 mL/kg of either colloid or crystalloid fluid

	Plasma volume expansion (mL, %)	Blood volume expansion (mL, %)	Plasma loss during fluid challenge (mL)	Average rate of plasma loss (mL/min)
Colloid				
End of fluid challenge	274 (+8.6%)	274 (+6.1%)	−6.3	−0.9
5 min later	264 (+8.3%)	264 (+5.9%)	−10.2	−1.5
Crystalloid				
End of fluid challenge	228 (+7.2)	228 (+5.1)	−51.6	−7.4
5 min later	164 (+5.2)	164 (+3.7)	−64.0	−9.1

**Fig. 5:**
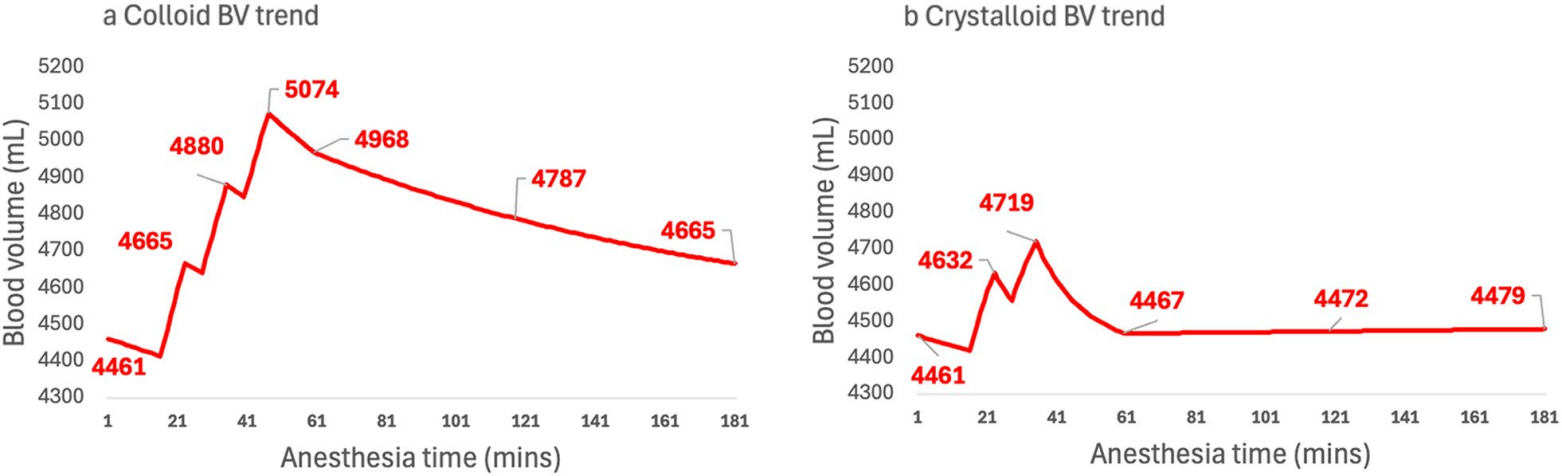
Exploratory analysis of the potential longer-term impact on BV of both choices of fluid and over sensitivity of the fluid responsiveness test when crystalloid is used. The attenuation of the crystalloid fluid bolus resulted in only 2 × 4 mL/kg fluid challenge being given (right panel) vs. 3 × 4 mL/kg for the colloid simulation (left panel). The resulting blood volume trend analysis shows a reduced peak post fluid challenge: 4719 vs. 5074 mL and lower blood volume trending throughout the operation. Patient details: Male, body weight 70 kg, age 60 years old, maintenance fluid rate 1.5 mL/kg/min with 300 mL of blood loss in the first hour

Figure 5 highlights the potential for longer-term blood volume trend impact from using crystalloid for fluid challenges. The simulation assumes a false negative fluid responsiveness result i.e. change in SV of <10% for the second crystalloid fluid challenge. This reduces the number of fluid challenges given from to 3 to 2 with crystalloid vs the situation where 3 colloid fluid challenges are given. At all points after the first bolus administration, the blood volume is considerably lower after crystalloid fluid challenges and at 3 h the resulting blood volume is still 350 mL lower than would be seen if colloid had been administered.

## Discussion

### Key findings

The primary hypothesis that the volume expansion resulting from multiple bolus infusion during surgery is dependent on the pharmacokinetic profile of the fluid used was confirmed by the kinetic model analysis. The plasma volume responses to a series of bolus infusions are gradually weakened when crystalloid fluid is used. Distribution fluid loss rates build up as the plasma volume expands and by the third fluid challenge two-thirds of the plasma volume expansion seen for the first fluid challenge is lost for patients placed in the flat recumbent position. In contrast, the plasma volume change for all the three HES fluid challenges is much more repeatable and declines by only 14% i.e. 4.7× less across the three fluid challenges. Additionally, the plasma volume peaks following crystalloid fluid challenge infusions were both lower and more variable than when colloid was used — confirming the secondary hypothesis. The magnitude of the crystalloid fluid losses looks to be clinically material, likely to negatively impact the diagnostic assessment of fluid responsiveness through monitoring the SV response. There is clearly the potential to be misled by the SV response to progressively reduced plasma volume changes seen in a sequence of crystalloid fluid challenges. The reduced hemodynamic response is likely to be interpreted as showing non-responsiveness i.e. a change in SV of less than 10%, but this conclusion might be erroneous due to fact that cardiac preload might not have been adequately increased.

Variations in the plasma volume response profile described above occur depending on body position and hemorrhage. The modifications of the kinetic parameters attributable to these factors were obtained by covariate analysis, which separated their influence from the kinetics that is valid for patients operated in the flat recumbent position. The head down position has most impact and slows distribution losses, which is probably due to gravitational forces, resulting in the infused fluid remaining in the plasma volume in a way like that of colloid fluid. After hemorrhage the peripheral space (the interstitium) but not the plasma volume remains filled, which slows down distribution and accelerates the return flow to the plasma. However, attenuation of the second and third bolus is still observed after the initial re-distribution of fluid.

### Interpretation

Unevenly distributed fluid always equilibrates between the plasma and interstitium by a combination of capillary leakage and lymphatic flow, but the process requires much longer time with a colloid compared to a crystalloid. For example, across the 7-min infusion period only 6 mL of a fluid challenge colloid bolus of 4 mL/kg (280 mL) is lost from the plasma volume. Thus, when the initial hemodynamic impact of the fluid is assessed, the plasma volume is expanded by close to 100% of the infused volume. At 5 min post fluid challenge, only a further 10 mL is lost, resulting in 94% of the infused volume is retained (Table 5). The slow distribution rate seen for colloid means that the blood volume expansion achieved for single or multiple sequential boluses will be repeatable and maintained, resulting in cardiac preload increase(s) that will consistently challenge the Frank-Starling Law of the heart.

By contrast, the plasma volume response to a crystalloid bolus is quite different, being dependent on both the infused volume and the difference in volume expansion between the plasma volume and the peripheral (interstitial) fluid space. Direct comparison with the colloid example shows that during the 7-min infusion period, 52 mL of fluid will be lost from the plasma volume, with a further 64 mL lost over the next 5 min, resulting in a much-reduced net change in the plasma volume (Table 5). Crystalloid is only 63% as effective as colloid up to the time when hemodynamic assessment is likely to be made, increasing the plasma volume by +5.2 vs. +8.3% for colloid and the blood volume by +3.7 vs. +5.9%. Therefore, a crystalloid bolus infusion will show poorer efficacy than a colloid in increasing and maintaining a change in the circulating blood volume.

This effect becomes more impactful for additional boluses as they are typically administered before the 30-min equilibration period for crystalloid fluid is completed. The increase of the plasma volume after the second and third bolus was only 51% and 36% as great as for the first one.

Moreover, measurement of SV made shortly after a crystalloid bolus are likely to be made on a “moving target” as the plasma volume/blood volume expansion is falling rapidly. This will not be the case with use of a colloid. The stronger plasma volume expansion in hypovolemic settings is due to the opposite disequilibrium, as the interstitium is then well hydrated while the plasma volume is not.

### Literature

Fluid responsiveness is dependent on an administered fluid bolus being large enough to challenge cardiac preload. Aya et al*.* [21] infused increasing volumes of crystalloid fluid (1, 2, 3, and 4 mL/kg) and found them to gradually increase the incidence of fluid responsiveness (20, 35, 45, and 65%); they concluded that 4 mL/kg should be used to adequately increase preload.

The fluid challenge volume should also be given quickly. Schmidt et al*.* [27] found that infusing 250 mL of a crystalloid or colloid fluid over 5 min was more effective, causing a greater increase in SV and MAP, than when delivered over 20 min. Messina et al*.* [28] similarly showed that the infusion time of a crystalloid bolus (4 mL/kg) affects the determination of fluid responsiveness. They also showed that the pharmacodynamic effect on flow variables fades rapidly — within 5 min of the end of the infusion.

A few previous studies suggest that the plasma volume response to sequential crystalloid boluses becomes progressively lessened/attenuated. In volunteers, the maximum plasma volume expansion from Ringer´s solution given in the hypervolemic state (2 h after HES) was only 68% of the expansion found when Ringer was given alone. [17] Simulations based on kinetic data showing attenuation of the plasma volume response to repeated short crystalloid infusions has been pointed out and illustrated in the past, but gained limited attention [8, 29].

László et al*.* compared the volumes needed when performing GDT with Ringer or HES in 30 patients undergoing flap surgery [30]. Ringer required 61% more bolus infusions and 43% more fluid, but only HES effectively increased cardiac index.

The SV response was not measured in this study. However, Prather et al*.* reported that the blood volume expansion decreased along with cardiac output after ending a large fluid infusion in dogs; the decline occurred much faster with crystalloid fluid than with a colloid or blood. [31] Norberg et al*.* found that both plasma dilution and cardiac output decreased within 5 min after a 20-min infusion of isotonic saline in volunteers [32], which is consistent with the loss of hemodynamic effect 5 min after a crystalloid fluid challenge reported by Messina et al. [28].

Li et al*.* used the same set-up as in the present study [20]. They induced general anesthesia and provided three successive bolus infusions of 3 mL/kg of either HES (N = 86) or Ringer (N = 25) over 7 min, followed by a free interval of 5 min during which SV was measured. The plasma volume expansion induced by Ringer amounted to 86%, 64% and 51% of the expansion found by HES. In comparison, simulations based on the present kinetic data yielded 81%, 65%, and 55%, respectively. Thus, real data confirm the proposed attenuation effect, and this clearly affected the SV responses. Induction of anesthesia decreased SV to 62% of baseline whereafter Ringer increased this fraction to 68% while HES raised it to 84%.

### Volume kinetics

Population pharmacokinetics is an industrial standard approach that we apply here on infusion fluids. Data from experiments performed during general anesthesia were consistently used because it is the typical scenario for GDF with fluid kinetics that clearly differs from the awake state. The diuretic response to volume expansion (*k*_10_) is only 10–20% of what is found in the awake state [11]. The return flow of distributed fluid from the peripheral space to the plasma amounts to 30–50% compared to the awake state due to inhibitory effects of general anesthetics on lymphatic pumping [33]. Hence, the capillary refill response to bleeding operates poorly during anesthesia, which maintains the attenuation of repeated bolus infusions on the plasma volume during hemorrhage (Fig. 3B).

A third effect consists of a MAP-dependent redistribution of interstitial fluid to the plasma during the first 20 min after anesthesia induction. Early inflow increases the plasma content of albumin, suggesting that the redistribution occurs via the lymph [34]. This redistribution was calculated off the present results by application of the covariates “0–10 min” and “10–20 min” in Table 2 as we believe it maintains cardiac preload rather than increasing it.

### Clinical implications

A dilemma occurs in terms of interpretation of SV response when the first bolus fluid indicates fluid responsiveness but the second, or third, bolus do not. This can either be due to the patient being really on the flat portion of the Frank-Starling curve, or else that attenuation of the plasma volume expansion makes the second bolus too small to adequately increase cardiac preload. The same dilemma would not happen if colloid fluid was used, as the plasma/blood volume expansion stemming from each bolus is more regular/repeatable meaning that attenuation of the expansion is a much smaller issue. When crystalloids are used, it is possible that this attenuation issue has negatively impacted the sensitivity and specificity and increased the grey zone area of uncertainty reported for the dynamic fluid responsive parameters PVI, PPV and SVV.

Equally important is that the hemodynamic measurement of SV performed after a fluid challenge will be affected by the rapid loss of fluid from the vascular compartment. Our model predicts that most of the plasma volume expansion effect of a crystalloid bolus is quite short lived. The distribution losses we report are quick enough to be the primary reason why the SV response to a fluid challenge has been reported to fall off so quickly. A reasonable steady state is not obtained until 30 min has passed since the last crystalloid bolus infusion ended, but to await full equilibration would make assessment of fluid responsive states and SV optimization difficult to apply clinically.

In theory, the important issue for attenuation is only that an acute disequilibrium has developed between the central and peripheral fluid volumes. It does not seem to matter much if the patient has equilibrated at a low (dehydrated) or high (hyperhydrated) level. For example, plots using kinetic data obtained for crystalloid fluid after furosemide-induced dehydration (1.5–2.0 L) show the same attenuation as in normovolemic patients [24].

The take-home message is that when using crystalloids, the volume used for the second bolus must be 50% larger than the first one. The third bolus be increased by an additional 25%. Three bolus infusions that each expand the plasma volume to the same degree requires 1280 mL in a patient weighing 70 kg if the 4 mL/kg recommended by Aya et al*.* [21] is used for the first bolus. The corresponding volume of colloid would amount to 735 mL. Using the same volume of crystalloid for each fluid challenge will result in an over estimation of fluid non-responsive states. Another important consequence of using crystalloid is that there will be a shorter duration hemodynamic impact compared to the use of colloids.

Exploratory analysis of the potential longer-term impact on the blood volume of both choice of fluid and over sensitivity of the test, resulting in an earlier diagnosis of fluid non-responsiveness (Fig. 5) gives an insight into how the shift to the use of crystalloid for fluid challenges may have resulted in the anesthetist having to manage a quite different i.e. lower blood volume, potentially barely above the initial post induction level. It is likely that this will result in a materially lower stroke volume throughout the operation, compared to using colloids in GDT protocols. It seems very possible that fluid challenges using crystalloid, compared to a colloid, or indeed a passive leg raise (whole blood infusion), are both pharmacokinetically and pharmacodynamically quite different.

Kinetic modeling raises the possibility that the recent shift to the use of crystalloids for the SV optimization component of GDT protocols has had the potential to impact the volume of fluid and use of supportive vasoactive and inotropic drugs used to maintain blood pressure, cardiac output and maintain hemodynamic stability. We cannot say if this move away from colloids has reduced the efficacy of GDT protocols used in more recent studies, but it is a factor that could be considered when evaluating these trials and in the design of future GDT study protocols.

Our analysis suggests that fluid challenges and the interpretation of fluid responsiveness would benefit from being more carefully standardized, not just in terms of volume and rate of administration, but also in terms of understanding the implications of the choice of fluid used. In the future, applied fluid pharmacokinetic analysis may find itself fulfilling a role in clinical practice, both to more precisely tailor fluid challenges and judge fluid responsiveness and also provide more guidance as to how to achieve additional precision in fluid and blood volume management in major surgery patients.

### Limitations

The patient data represent all existing studies of anesthetized patients where volume kinetic data has been retrieved that are not confounded by vasopressor therapy, major surgical hemorrhage, deliberate dehydration or deliberate blood withdrawal, or severe disease. Hence, the simulations were based on data sets from 103 anesthetized patients studied in the same way, all performed or monitored by RGH, but with other questions than the one raised here. These studies were all associated with minor hemorrhage that was corrected individually in a way that caused slight depression of the plasma dilution-time curve.

The inclusion of a diverse set of operations represents a wish to cover different clinical settings, which also means that the results are widely applicable. Kinetic heterogeneity was handled by covariate analysis, where influences of the distribution rate constant, *k*_12_, was shown to occur from altered body positions (Table 2). This is the most essential parameter determining the shape of the plasma volume curve when short bolus infusions of crystalloid fluid are administered (for a colloid, it is the intravascular persistence time). It is of note that no covariance was found between *k*_12_ and the age, body weight, sex, infused volume, or arterial pressure. However, simulation of a predetermined hemorrhage was performed based on kinetic data from blood withdrawal in awake volunteers, which makes this part of the study speculative.

The kinetic data for HES was taken from a previous study of awake volunteers. No similar data for HES derived during general anesthesia is available. However, colloid fluid does not have a distribution phase that is detectable by volume kinetics [22, 23]. Therefore, the intravascular persistence time is the kinetic parameter determining the shape of the plasma volume curve when a series of bolus infusions of colloid fluid are given in sequence. A recent study of gelatin (Gelofusine) showed a virtually identical intravascular persistence time during vascular surgery as HES did, which suggests that the present simulations and arguments are applicable to Gelofusine as well [26].

The baseline volume of *V*_c_ is called the plasma volume although this is a functional volume. However, the approximation is reasonable given that *V*_c_ was 3.3 L for males (77 kg, Hb 116 g/L) and 2.4 L for women (63 kg, Hb 119 g/L; Table 2), which does not deviate much from the expected plasma volume in these patients. A larger *V*_c_ than expected is a sign of vasodilatation, which was not a prominent feature in the present evaluation. “Stress relaxation” of the vascular system might occur following more vigorous fluid loading [31], but vasodilatation in the present study would be more likely due to general anesthesia. However, vasodilation could have been counteracted by other factors, such as endogenous catecholamines. No exogenous catecholamines were administered to the studied patients.

The infused fluid volumes in the studies used to derive the kinetic parameters used for simulation were larger than the volume given during a single series of bolus infusions, while it agreed better with two series of boluses. However, the kinetic model is built on proportionality between volume expansion and capillary leakage, which means that leakage occurs half as fast if the volume expansion is half as great. Zero-order or receptor-binding models have for long been ruled out as applicable for volume kinetics; the only exception from mono-exponential dependence seems be at hand when fluid accumulates in the “third fluid space”. However, infusion of > 1.5 L is required for the “third space” to open for fluid accumulation [18].

The kinetic analysis is based on data where no fluid was infused during induction of general anesthesia. The distribution of fluid infused during the induction phase is greatly affected by the arterial pressure during approximately 20 min [35]. If no fluid is given the vasodilatation is compensated by increased lymphatic flow [34] which, in the present study, was calculated off the present kinetic data by covariate analysis. Those who infuse fluid during the onset of anesthesia should consider the values obtained after constant-rate infusion of fluid in Table 3. However, as full volume equilibration requires 30 min to be completed for crystalloid fluid, and providing a bolus at an earlier stage always activates the attenuation effect demonstrated here.

## Conclusions

The simulations show that the plasma volume response to three successive bolus infusions of crystalloid fluid gradually becomes smaller. This can make clinicians erroneously believe that a patient is volume optimized after a second or first bolus although the real reason is that the increase of cardiac preload is inadequate. The infused volume must be increased if a constant effect of the plasma volume is to be obtained. The issue of attenuation is not shared with colloid fluid.

## Supplementary Information

Additional file 1.

## Data Availability

The original data is available as Supplementary File.xls.
